# Severe Intrusive Luxation of a Permanent Maxillary Central Incisor Managed Through an Interdisciplinary Approach Using Orthodontic Extrusion and Endodontic Therapy: A Five-Year Follow-Up

**DOI:** 10.7759/cureus.109800

**Published:** 2026-05-28

**Authors:** Meenakshi Chandel, Teh M Chou, Priyanka Agarwal, Pooja Gupta, Aman Singh, Pradeepti Singha

**Affiliations:** 1 Pedodontics and Preventive Dentistry, Faculty of Dental Sciences, Institute of Medical Sciences (IMS), Banaras Hindu University (BHU), Varanasi, IND; 2 Orthodontics, Sukh Sagar Medical College and Hospital, Jabalpur, IND; 3 Pediatric and Preventive Dentistry, Index Institute of Dental Sciences, Indore, IND

**Keywords:** dental injury, luxation injuries, orthodontic extrusion, pediatric trauma, severe intrusion

## Abstract

Intrusive luxation of a permanent maxillary central incisor is an uncommon yet highly challenging dental injury, often associated with extensive periodontal ligament (PDL) damage, pulpal necrosis, and an increased risk of root resorption.

This case report details the comprehensive management of a severely intruded maxillary central incisor in a nine-year-old patient using controlled orthodontic extrusion. Clinical and radiographic evaluation confirmed significant apical displacement without evidence of crown or root fracture. Given the depth of intrusion and the stage of root development, orthodontic extrusion was chosen as a biologically conservative and predictable approach to reposition the tooth and support periodontal healing. Endodontic therapy was subsequently performed to prevent inflammatory root resorption. Over a five-year follow-up period, the tooth showed stable positioning, healthy periodontal support, and favourable aesthetic and functional outcomes.

This case demonstrates the reliability of orthodontic extrusion as a minimally invasive and effective treatment modality for severe intrusive luxation injuries in permanent incisors.

## Introduction

Traumatic dental injuries (TDIs) account for approximately 5% of all bodily injuries and remain the most commonly reported trauma among adolescents and young adults [1]. Epidemiological studies indicate that about one-third of adults and one-quarter of school-aged children experience trauma to their permanent teeth, with most incidents occurring before the age of 19 [2].

Intrusive luxation is the displacement of a tooth in an apical direction into the alveolar bone or socket. This injury most frequently occurs in the maxillary anterior region and is often associated with alveolar bone fractures, as well as pulpal and periodontal damage [1]. Traumatic injuries to permanent anterior teeth also pose significant psychological and emotional concerns for affected children and their parents [3].

Accurate clinical and radiographic assessment is essential for determining the direction and extent of displacement in intrusive injuries. The subsequent treatment plan and prognosis depend on multiple factors, including patient age, type of dentition, stage of root development, and the depth and duration of intrusion [4]. Treatment options for intrusive luxation include spontaneous re-eruption, orthodontic repositioning, and surgical repositioning [1,4].

Spontaneous re-eruption is often considered for mildly or moderately intruded teeth with open apices. Orthodontic repositioning with light forces is recommended for severely intruded teeth, typically when intrusion exceeds 7 mm. Surgical repositioning is indicated when immediate access to the pulp is required or when the intrusion is extensive.

The timing of orthodontic extrusion is critical. Initiating treatment immediately can help prevent ankylosis and enable early endodontic access, though it may also carry risks, including root resorption and alveolar bone loss. Conversely, delayed extrusion allows periodontal tissues to stabilise but increases the risk of ankylosis [5]. Current International Association of Dental Traumatology (IADT) guidelines recommend initiating orthodontic repositioning at approximately four weeks for teeth with open apices and around eight weeks for those with complete root development. However, these timeframes are clinical guidelines and must be adapted to individual patient factors, including signs of ankylosis, periodontal status, and the urgency of endodontic access [4-6].

This case report discusses the successful management of a severely intruded permanent maxillary central incisor through orthodontic extrusion. Current evidence supports this approach as the preferred option for severe intrusive luxation in permanent teeth with closed apices, as it applies controlled, biologically appropriate forces while minimising further damage to the compromised periodontium [4,7,8]. Notably, the present case is clinically significant because it involves a nine-year-old patient with a closed apex, an atypical finding for this age, accompanied by severe intrusion of more than 7 mm. This combination substantially increases the risk of pulpal necrosis, root resorption, and ankylosis, making treatment planning particularly challenging and the documentation of long-term outcomes especially valuable [3,9-11].

## Case presentation

**Figure 1:**
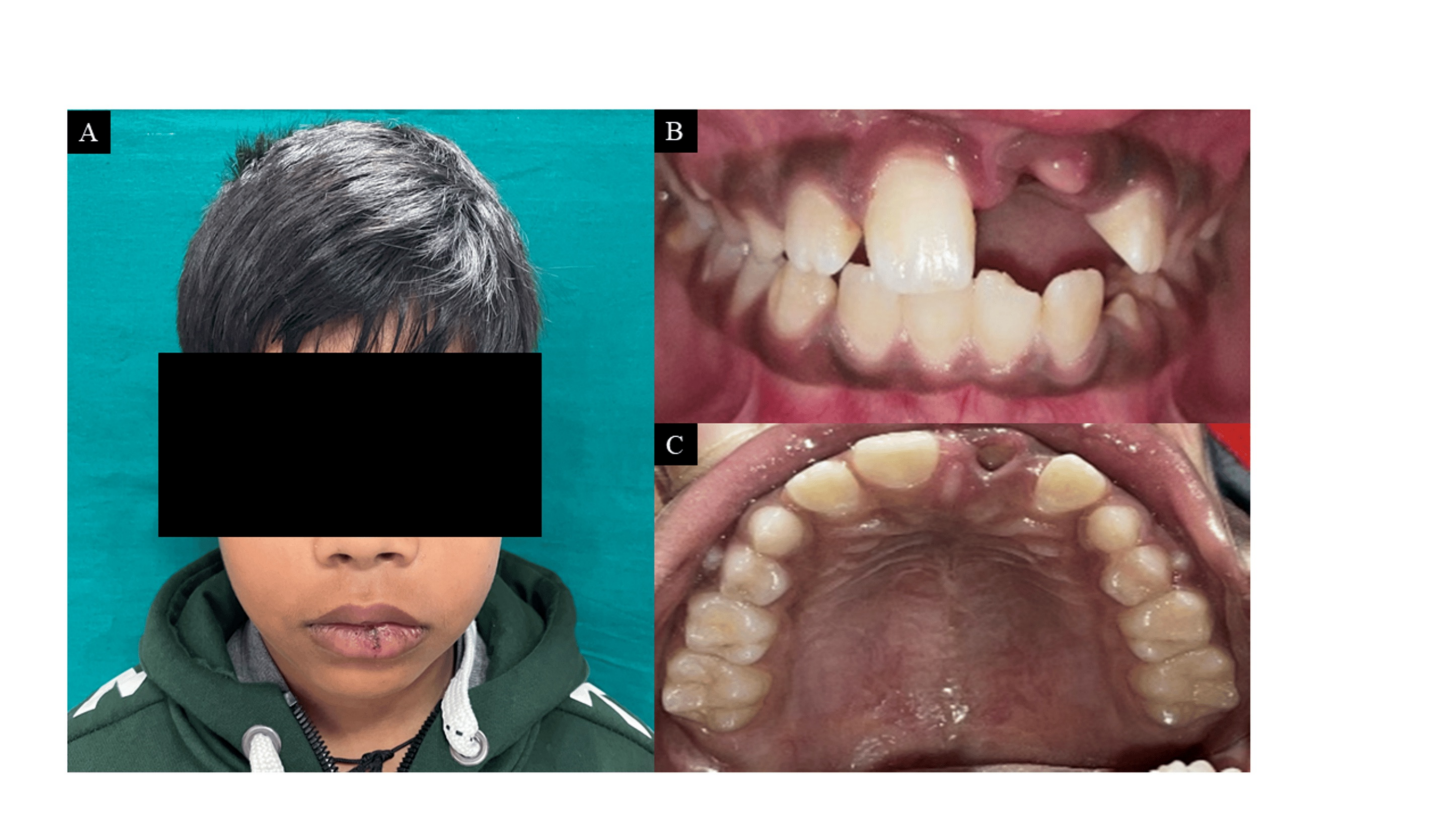
Preoperative clinical presentation. A) Extraoral frontal view showing no facial asymmetry or soft-tissue swelling. A healing laceration is visible on the lower lip, consistent with the reported mechanism of injury. B) Intraoral frontal view demonstrating severe intrusive luxation of the maxillary left central incisor (tooth 21), with marked apical displacement and disruption of the gingival contour relative to adjacent teeth. C) Maxillary occlusal view confirming the extent of intrusion. The patient provided written and signed consent, allowing publication of this identifiable facial image in an open-access journal.

A nine-year-old boy presented to the Pediatric and Preventive Dentistry unit approximately 22 hours after a bicycle accident, with a chief complaint of his upper front tooth being pushed inward (Figure 1). He reported initial bleeding that stopped after rinsing, with localised pain. His medical and vaccination history was unremarkable, and there were no signs of head injury or systemic involvement.

**Figure 2:**
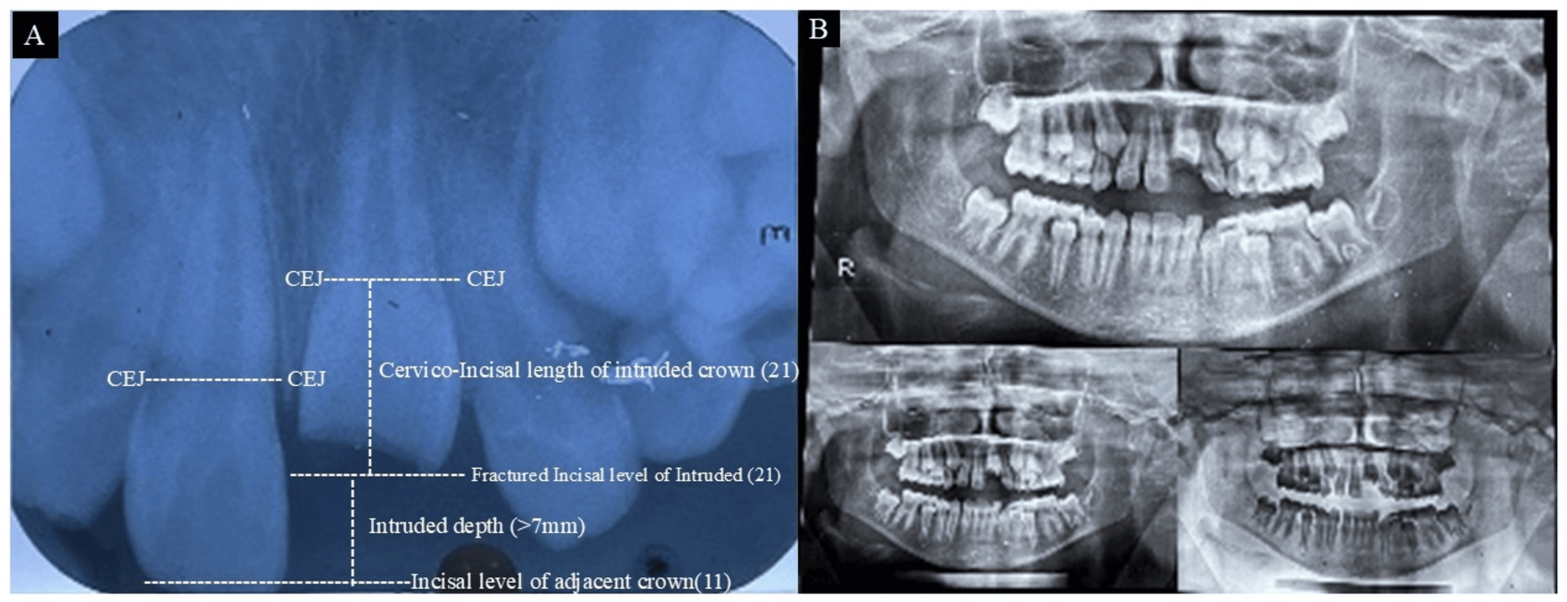
Preoperative radiographic assessment. A) Intraoral periapical radiograph demonstrating complete intrusion of the maxillary left central incisor, with apical displacement, narrowing of the periodontal ligament space, and a closed apex. No root or alveolar bone fractures are evident. B) Panoramic radiograph confirming the extent of intrusion and the absence of associated dentoalveolar fractures.

Intraoral examination revealed severe intrusive luxation (>7 mm) of the maxillary left central incisor (21), a lower-lip laceration, and an Ellis Class I fracture of the lower left central incisor (Figure 1). Radiographs, including two periapical views and a panoramic image, confirmed complete intrusion with closed apices, narrowing of the periodontal ligament (PDL) space, and no evidence of root or alveolar bone fractures (Figure 2). The depth of intrusion was assessed both clinically and radiographically. Clinically, the degree of displacement was estimated by comparing the incisal edge of the intruded tooth with the adjacent maxillary right central incisor and the contralateral gingival margin. Radiographically, the extent of apical displacement was measured on the periapical radiograph using the cementoenamel junction of the adjacent incisor as a reference landmark, confirming intrusion of more than 7 mm [3,4,12].

At baseline, clinical parameters were recorded as follows: periodontal probing depths around adjacent teeth were within normal limits (≤3 mm); pulp sensibility testing of the intruded tooth showed no response to cold (Endo Ice), consistent with pulpal disruption; percussion produced a high-pitched metallic sound, suggesting early ankylosis; and tooth mobility was not assessable due to the degree of intrusion. The adjacent teeth showed normal sensibility and mobility.

Soft-tissue injuries were cleaned, and the patient was monitored for spontaneous re-eruption over two weeks. Although IADT guidelines recommend initiating orthodontic repositioning at approximately eight weeks for teeth with closed apices, the decision to proceed earlier in this case was based on the severity of intrusion (>7 mm), the absence of spontaneous tooth movement within the first two weeks, and the elevated risk of ankylosis with prolonged delay in closed-apex cases. This short observation window aligns with emerging evidence suggesting that earlier intervention for severe closed-apex intrusions may reduce the risk of ankylosis without significantly increasing the risk of root resorption [4,5,13]. Consequently, orthodontic repositioning was initiated using a fixed multibracket appliance with elastic traction.

**Figure 3:**
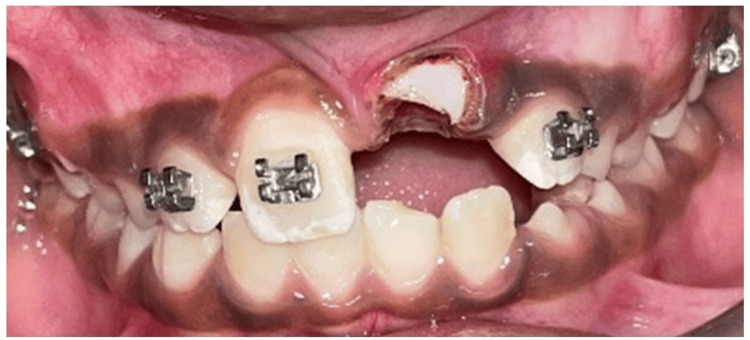
Surgical exposure and initiation of orthodontic extrusion. Intraoral frontal view after labial and palatal gingivectomy with electrocautery to expose the crown of the intruded tooth, and facilitate endodontic access and bracket placement.

**Figure 4:**
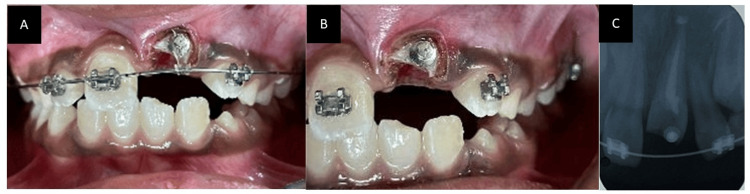
Orthodontic extrusion phase. A & B) Intraoral frontal views showing the application of continuous orthodontic traction with a segmental fixed appliance (MBT, 0.022" slot) and a 0.016" NiTi archwire, using elastomeric traction. Adjacent maxillary anterior teeth serve as anchorage units. C) Periapical radiograph during the extrusion phase confirming progressive coronal repositioning of the intruded incisor within the alveolar socket, with maintained root integrity and a developing periodontal ligament space.

Given persistent pain and the increased risk of pulpal necrosis in mature teeth, endodontic therapy was planned concurrently. A gingivectomy was performed labially and palatally using electrocautery to expose the crown and facilitate root canal access (Figure 3). A calcium hydroxide (Ca(OH)₂) dressing was placed for one week. During the same appointment, a segmental fixed appliance (MBT, 0.022" slot) was bonded, and light orthodontic traction was applied with a 0.016" NiTi wire. A lingual button was placed on the intruded tooth, and brackets were bonded to adjacent teeth. Elastomeric modules were replaced every two weeks to maintain consistent extrusive forces (Figure 4). Light, continuous extrusive forces of approximately 20-40 g were applied, consistent with recommended thresholds for orthodontic tooth movement after periodontal injury. Anchorage was derived from the adjacent maxillary right central incisor and both lateral incisors, which were incorporated into the segmental appliance. Care was taken to avoid heavy forces that could exacerbate PDL damage or precipitate external root resorption [5,8,14].

**Figure 5:**
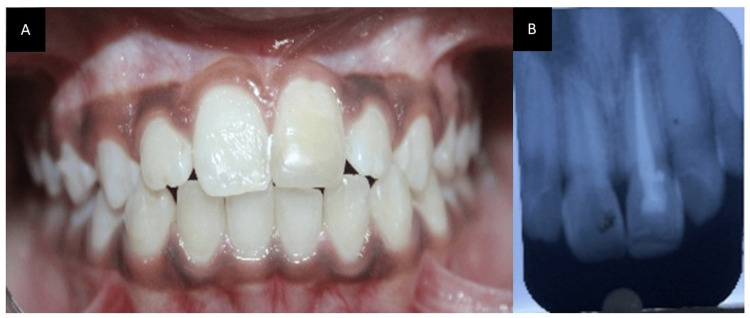
Post-extrusion status and endodontic outcome. A) Intraoral frontal view after completion of orthodontic extrusion, showing satisfactory alignment of tooth 21, with an acceptable gingival contour and aesthetics. B) Periapical radiograph confirming root canal obturation, re-establishment of the periodontal ligament space, and absence of pathological root resorption or periapical pathology.

Ca(OH)₂ dressings were replaced at one week and again at two weeks. Follow-up visits were scheduled every 15 days. The arch-wire sequence progressed from 0.018" and 0.018×0.025" NiTi to 0.019×0.025" stainless steel. Step-down bends were incorporated into the stainless-steel wire to ensure adequate traction without interfering with the segmental appliance. The root canal was obturated one month after the initiation of treatment (Figure 5). Root canal treatment was performed under rubber dam isolation. Working length was determined using an electronic apex locator and confirmed radiographically. Biomechanical preparation was carried out using a crown-down technique with rotary NiTi instruments. Irrigation was performed with a 2.5% sodium hypochlorite solution, followed by a final rinse with a saline solution containing 17% ethylenediaminetetraacetic acid (EDTA). Ca(OH)₂ was used as an intracanal medicament for approximately four weeks between appointments to suppress inflammatory root resorption. Root canal obturation was performed using cold lateral compaction of gutta-percha with AH Plus resin sealer (Dentsply Sirona, Bensheim, Germany), one month after the initiation of treatment. The access cavity was restored with composite resin [4,7,15].

Orthodontic extrusion occurred gradually at approximately 1-2 mm per month. Throughout treatment, no signs of root resorption or soft-tissue complications were observed. After 26 weeks, the intruded tooth had fully returned to the level of the adjacent central incisor. After active orthodontic treatment, clinical re-evaluation showed normal mobility (Grade 0), a dull percussion sound indicating the absence of ankylosis, and periodontal probing depths within normal limits bilaterally. A positive pulp sensibility response was not anticipated, given the completed endodontic therapy. The active orthodontic phase lasted seven months, followed by a six-month retention period. The outcome showed excellent alignment, occlusion, and functional stability, and radiographs showed no evidence of external root resorption (Figure 5).

**Figure 6:**
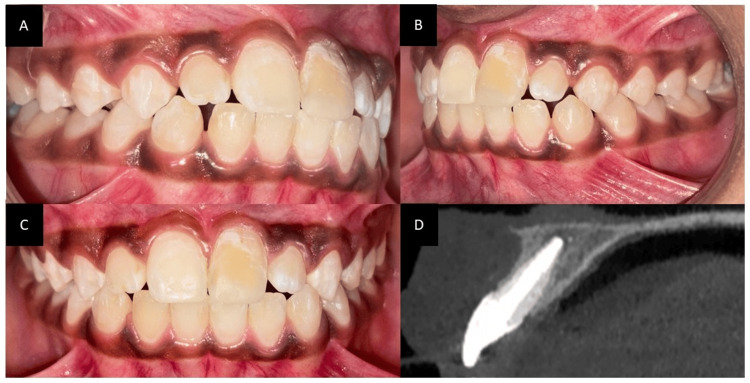
Five-year follow-up. A-C) Intraoral clinical photograph showing stable alignment, healthy gingival architecture, and a satisfactory long-term aesthetic outcome. D) CBCT cross-sectional view confirming maintenance of alveolar bone integrity, absence of external root resorption, and favourable periapical status at five-year follow-up. CBCT: cone-beam computed tomography

The patient was lost to periodic interim recall despite repeated attempts to re-establish contact; consequently, structured follow-up at the recommended six-month and annual intervals was not possible. However, the patient presented voluntarily at five years post-treatment. At the five-year follow-up appointment, outcomes were systematically assessed across the following parameters: (1) symptoms: the patient reported no pain, sensitivity, or functional difficulty; (2) mobility: Grade 0, consistent with normal periodontal support; (3) percussion: dull tone, with no metallic, high-pitched sound suggestive of ankylosis; (4) pulp status: the tooth was root canal treated; therefore, sensibility testing was not applicable; (5) crown discoloration: absent; (6) periodontal probing depths: within normal limits (≤3 mm) at all six sites around tooth 21, with no bleeding on probing; (7) gingival recession: absent, with gingival margin symmetry maintained relative to the contralateral incisor; (8) occlusal stability: satisfactory intercuspation with no fremitus or premature contacts; (9) periapical status: radiographically within normal limits (Figure 6); (10) root resorption: absent on cone-beam computed tomography (CBCT) (Figure 6); (11) alveolar bone integrity: maintained on CBCT with no vertical bone loss (Figure 6). These findings, although limited to a single long-term recall rather than sequential follow-up, are consistent with a favourable long-term outcome. The entire chronological timeline of clinical events from initial trauma presentation to five-year follow-up has been summarised in Table 1.

**Table 1:** Chronological timeline of clinical events from initial trauma presentation to five-year follow-up.

Timepoint	Event
Day 0	Bicycle accident - traumatic intrusion of tooth 21.
Day 1 (22 hours post-trauma)	First presentation: clinical and radiographic assessment; soft tissue management; composite restoration on tooth 31.
Week 2	Case reviewed; no spontaneous re-eruption observed; decision to initiate orthodontic extrusion.
Week 2-3	Gingivectomy (electrocautery); Endodontic access opening performed, followed by pulp removal and calcium hydroxide dressing placement; segmental fixed appliance bonded; 0.014" NiTi wire placed; orthodontic traction initiated the next day.
Week 3-4	Calcium hydroxide dressing was replaced 2 weeks after placement.
Month 1	Root canal obturation completed.
Months 1-7	Active orthodontic extrusion phase; archwire sequence progressed (0.014" NiTi → 0.016" NiTi → 0.018" NiTi → 0.018×0.025" NiTi → 0.019×0.025" SS); elastomeric modules replaced every 2 weeks.
Month 7 (26 weeks)	Full extrusion achieved - tooth 21 at the level of the adjacent central incisor.
Year 5	Final follow-up: clinically asymptomatic, no radiographic pathology on cone-beam computed tomography (CBCT); stable periodontal and periapical status confirmed.

## Discussion

In dental traumatology, intrusive luxation is considered the most severe form of dental trauma due to extensive damage to the PDL, alveolar bone, cementum, and the pulpal neurovascular supply. This case describes the management of a severely displaced permanent maxillary central incisor (>7 mm) in a nine-year-old child with a closed apex, treated with orthodontic extrusion and followed for five years. These cases carry a guarded prognosis, particularly when root development is complete [5].

The degree of displacement and the stage of root development are critical determinants of healing after intrusive luxation [6]. In this case, intrusion exceeded 7 mm, classifying it as severe. Teeth with closed apices have limited capacity for pulpal revascularisation because the apical blood supply is irreversibly disrupted, leading to a high incidence of pulp necrosis. Consequently, early endodontic intervention is recommended to prevent inflammatory root resorption, which remains one of the most common complications following intrusive injuries [9].

According to the IADT guidelines, treatment options for intruded permanent teeth include spontaneous re-eruption, surgical repositioning, and orthodontic extrusion, with the choice depending on the extent of intrusion and root maturity [4]. Spontaneous re-eruption is primarily indicated for teeth with incomplete root development and mild to moderate intrusion. However, in cases of severe intrusion and closed apices, spontaneous re-eruption is unpredictable and carries a higher risk of ankylosis and marginal bone loss [6]. Therefore, this approach was not considered appropriate for the present case.

Orthodontic extrusion was chosen for its biological advantages and favourable long-term outcomes. Gradual orthodontic repositioning enables controlled tooth movement while minimising further trauma to the damaged PDL and surrounding alveolar bone [8]. Compared with surgical repositioning, orthodontic extrusion has been associated with a lower risk of replacement resorption and ankylosis, particularly in growing patients [16]. In the present case, orthodontic forces enabled the successful repositioning of the intruded incisor, with satisfactory periodontal healing and preservation of alveolar bone contours.

The diagnosis and long-term assessment of this case were greatly aided by CBCT. CBCT enabled precise assessment of the extent of intrusion, the spatial relationship of the root to the alveolar bone, and early detection of root resorption or ankylosis, which may not be evident on conventional radiographs [12,16,17]. Over the five-year follow-up, CBCT findings confirmed stable periodontal healing, absence of progressive root resorption, and maintenance of alveolar bone integrity.

Long-term follow-up is essential after intrusive luxation, as complications such as ankylosis and replacement resorption may develop years after the initial injury. A notable limitation in the present case was the absence of regular follow-up and interim clinical and radiographic records. This limited the ability to monitor healing dynamics and detect early complications such as inflammatory root resorption or ankylosis. However, the five-year follow-up findings showed a clinically asymptomatic tooth with normal mobility and no radiographic pathology, indicating favourable periodontal and periapical healing.

The satisfactory aesthetic and functional outcomes further support the use of orthodontic extrusion as a conservative, biologically sound treatment for severe intrusive luxation injuries.

## Conclusions

This case report describes a favourable clinical outcome after interdisciplinary management of severe intrusive luxation of a permanent maxillary central incisor in a nine-year-old patient with a closed apex. The successful outcome was achieved through early diagnosis, adherence to IADT evidence-based guidelines, controlled orthodontic extrusion with light, continuous forces, timely endodontic intervention with Ca(OH)₂ to prevent inflammatory root resorption, and long-term clinical monitoring. These findings suggest that orthodontic extrusion may be a conservative and biologically sound option in carefully selected cases of severe intrusive luxation; however, given the inherent limitations of a single case report, broad generalisations about outcome predictability across all such presentations should be made with caution.

The five-year clinical and radiographic follow-up findings, including the absence of root resorption, stable periodontal support, and satisfactory occlusal and aesthetic outcomes, are encouraging and support the value of this interdisciplinary approach. However, the lack of structured interim follow-up at recommended intervals is a limitation of this report, and late-onset complications cannot be entirely excluded based on a single long-term recall. Future prospective case series with standardised radiographic monitoring protocols and consistent follow-up at defined intervals are needed to more reliably define the long-term prognosis of orthodontic extrusion in severe closed-apex intrusive luxation injuries.
